# *Staphylococcus schleiferi*: An Uncommon Pathogen in an Immunocompetent Adolescent with Chronic Osteomyelitis

**DOI:** 10.1155/2023/9597582

**Published:** 2023-10-12

**Authors:** Maria Celeste Ruiz Holgado, Joanna Iris Depasupil, Michael Stracher, Esra Fakioglu, Lily Q. Lew

**Affiliations:** ^1^Department of Pediatrics, Flushing Hospital Medical Center, 4500 Parsons Blvd, Flushing, NY 11355, USA; ^2^Department of Orthopedics, Jamaica Hospital Medical Center, 8900 Van Vyck Expressway, Jamaica, NY 11418, USA

## Abstract

*Staphylococcus schleiferi* represents an uncommon pathogen in human infections. As a veterinary pathogen, *S. schleiferi* causes canine ear and skin infections. Chronic osteomyelitis is a progressive process characterized by bone destruction and the formation of sequestrum. It may be a sequela of untreated or undertreated acute osteomyelitis or septic arthritis. Descriptions detailing the pathogenicity and virulence of *S. schleiferi* osteomyelitis were limited to a few case reports. Among the three reported cases of *S. schleiferi* osteomyelitis, immunosuppression, malignancy, and recent surgical procedures were comorbidities. Compared to those who are immunosuppressed, immunocompetent individuals are generally not susceptible to uncommon microorganisms. Early detection of osteomyelitis, aggressive appropriate prolonged antimicrobial treatment and a multidisciplinary approach contribute to optimal recovery. We report the first case of *S. schleiferi* chronic osteomyelitis in an immunocompetent adolescent.

## 1. Introduction

Unlike *Staphylococcus aureus, Staphylococcus schleiferi* represents an uncommon causative pathogen of osteomyelitis. Osteomyelitis is typically classified as either acute or chronic depending on the duration of symptoms. Chronic osteomyelitis is a progressive process involving the formation of an abscess, vascularized necrotic bone and the development of a sinus tract [1]. Although *S. schleiferi* differs from *S. aureus* in many microbiological ways, its identification should not be considered a contaminant [2]. Of the two known distinct subspecies described, coagulase-negative *S. schleiferi* subsp. schleiferi accounts for most of the infections in humans as opposed to coagulase-positive *S. schleiferi* subsp. coagulans [3]. Routine microbiology diagnostic protocols may misidentify *S. schleiferi* as *S. aureus* due to their similarity. A few case reports in humans illustrate the pathogenicity and virulence of *S. schleiferi* [4]. Exposure to infected dogs as a source of infection was in the majority of those reports [4]. Treatment strategies for *S. schleiferi* osteomyelitis should include aggressive drainage of abscesses, debridement of necrotic bone, and the use of appropriate antibiotic therapy based on bone cultures similar to the guidelines for *S. aureus* osteomyelitis [1]. With a multidisciplinary approach and prolonged antibiotic therapy, optimal recovery can be expected. Nevertheless, it is prudent to monitor *S. schleiferi* as an emerging zoonotic pathogen, to observe for antimicrobial resistance and to track the potential of nosocomial acquisition and transmission. We describe herein such a case of *S. schleiferi* chronic osteomyelitis in an otherwise healthy adolescent.

## 2. Case

**Figure 1:**
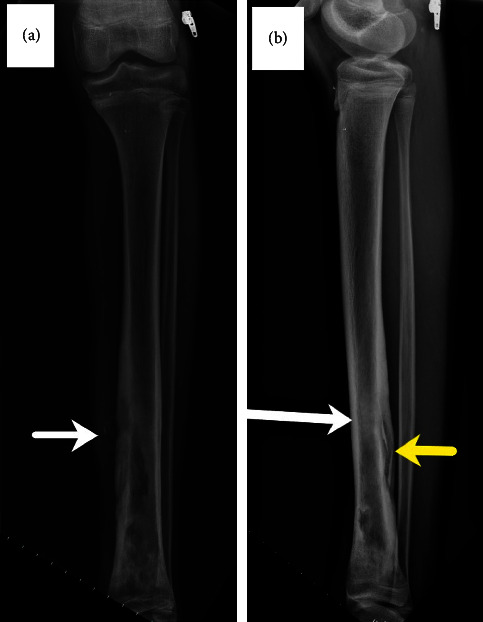
Plain radiographs of the left tibia showed osseous destruction throughout on (a) anteroposterior view and (b) lateral view with the sinus tract (white arrow), sequestrum, and surrounding involucrum (yellow arrow).

**Figure 2:**
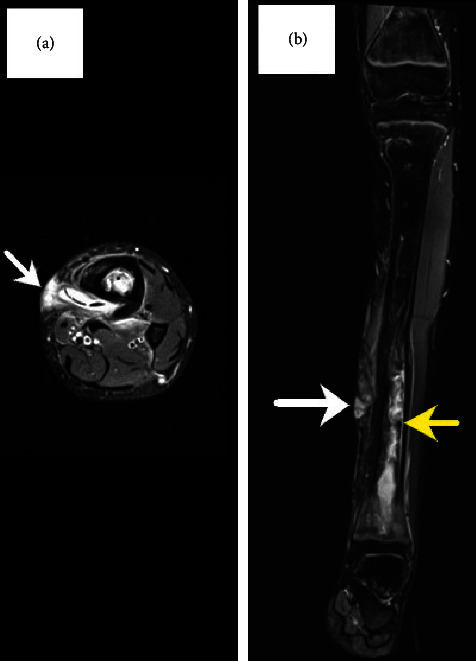
Magnetic resonance imaging (MRI) with gadolinium enhancement of the left leg on (a) transverse view and (b) coronal view demonstrating intramedullary edema (yellow arrow) and the sinus tract (white arrow).

A previously healthy 16-year-old male was admitted for management of a left lower tibia recurrent draining sinus. He sustained injury to the medial aspect of his left lower extremity after jumping off a four-foot wall in an urban construction site located in his native country of Jamaica one year prior to admission. Within a few days of his injury, he was admitted to a local hospital for swelling of the left ankle, an open wound, and fever. He underwent a surgical procedure and received three weeks of antibiotic treatment for reported left tibia fracture and infection. One week after hospital discharge, he immigrated to the United States. Four months after his arrival, he consulted his primary care physician because of a purulent draining sinus. He was prescribed trimethoprim-sulfamethoxazole (TPX-SMX) 160–800 mg twice a day for ten days to treat *S. aureus*. Another course of the same antibiotic was prescribed when drainage recurred pending surgical consultation. His household included two younger siblings and four domesticated dogs. At admission, there was a small draining sinus anteriorly in the distal third of his left lower extremity without erythema or swelling. He was afebrile. Laboratory findings included a leukocyte count of 4.3 × 10^9^/L (normal 3.6–11.0), hemoglobin 132.0 grams/L (normal 108.0–160.0), C-reactive protein (CRP) < 1.0 mg/L (normal <5.0), and an erythrocyte sedimentation rate (ESR) of 25 mm/hr (normal 0–14). The plain radiograph of the left tibia and ankle showed chronic deformity distally with lucency laterally and cortical irregularity along the dorsal aspect of the distal tibia, Figure 1. Magnetic resonance imaging (MRI) of the left leg demonstrated cortical thickening of distal half of the tibia, sinus tract, and bone marrow edema consistent with chronic osteomyelitis, Figure 2.

## 3. Hospital Course

Surgical intervention included excision of the sinus tract, debridement of necrotic bone, and placement of antibiotic impregnated beads. Bone cultures were positive for *S. schleiferi* subsp. coagulans resistant to penicillin. He was treated with vancomycin (60 mg/kg/day intravenously divided every 8 hours) for two weeks before transitioning to oral clindamycin (300 mg every 6 hours) based on the susceptibility pattern for a total of six weeks. CRP of <1.0 mg/L and ESR of 4 mm/hr were both normal three months postoperatively. He continued to be asymptomatic at one-year follow up.

## 4. Discussion

*S. schleiferi* represents an uncommon pathogen in human infections [4]. In veterinary medicine, the microorganism causes skin and ear infections in dogs [5]. Two subspecies of *S. schleiferi* have been described: subsp. schleiferi by Freney et al. in 1988 [6] and subsp. coagulans by Igimi et al. in 1990 [7]. Of the two subspecies, *S. schleiferi* subsp. schleiferi is more commonly associated with human infections, specifically of wounds, surgical sites, osteomyelitis, and bacteremia [4, 8]. In clinical settings, subspecies identification is typically not performed since treatment depends on antibiograms and the location of the infection. Due to similarities to *S. aureus*, reports suggested possible underrecognized, underestimated, and underreported incidence of *S. schleiferi* [9]. Methicillin-resistant *S. schleiferi* and nosocomial acquisition have been reported [10]. Except for a few sporadic case reports, little is known of pathogenicity and virulence [11]. To date, three case reports of *S. schleiferi* osteomyelitis have been found in the literature, Table 1. Immunosuppression, malignancy, and recent surgical procedures were comorbidities in those cases [4]. We are reporting the first case of *S. schleiferi* chronic osteomyelitis in an otherwise healthy adolescent.

Acute osteomyelitis typically presents with symptoms within two weeks of bone injury, whereas chronic osteomyelitis represents a progressive condition, occurring over weeks, leading to the development of necrotic bone or sequestra. Symptoms specific to chronic osteomyelitis include draining or persistent sinus tracts, poor wound healing, and absent constitutional symptoms. Besides using duration of symptoms, and histopathological findings to classify osteomyelitis, the Cierny–Mader classification is often applied to adults with chronic osteomyelitis. This classification incorporates the anatomical type and physiological class [12]. Our case would be classified as IIIA when using Cierny–Mader classification, III to indicate a localized lesion with medullary and cortical involvement and A for host with a good immune system.

In chronic osteomyelitis, the abscess that forms as a result of inflammation and infection leads to the development of vascularized necrotic bone known as sequestrum and potentially, a sinus tract [12]. The prognosis and outcome of chronic osteomyelitis depend on early recognition of symptoms, rapid diagnosis, drainage of abscesses, debridement of necrotic bone, prolonged antimicrobial treatment, and a multidisciplinary approach. A history of trauma, open fracture, surgical trauma, chronic open wound, and an immunocompromised host are predisposing factors of chronic osteomyelitis [12]. Supporting laboratory tests to diagnose osteomyelitis such as leukocytosis, elevated ESR, and elevated CRP lack specificity and are not diagnostic. However, serial CRP levels may be helpful in monitoring clinical response and treatment [13]. Imaging studies including the plain radiograph and MRI confirm the clinical diagnosis. The plain radiograph can identify fracture, periosteal elevation, and hardware in addition to sequestrum and involucrum in advanced cases. MRI, the imaging study of choice, has the highest sensitivity and specificity in detecting osteomyelitis [14]. Fluorodeoxyglucose positron emission tomography (FDG-PET) is the most accurate in diagnosing chronic osteomyelitis and limited by its cost and availability [14]. Management of chronic osteomyelitis includes surgical drainage, debridement, and sequestrectomy followed by prolonged aggressive antibiotic therapy. The bone culture and histopathologic examination will positively confirm the diagnosis of chronic osteomyelitis [12]. Special culture techniques may be necessary to identify some organisms. The sensitivity pattern can guide antibiotic therapy. A multidisciplinary approach has improved prognosis and outcome of chronic osteomyelitis [12].

Our patient developed swelling, an open wound, and fever within days after the fall suggestive of acute osteomyelitis. By history, he underwent a surgical procedure and three weeks of antibiotic treatment. The recurrent draining sinus over a course of several months and having necrotic bone were consistent with chronic osteomyelitis. The pathogen entered most likely through the original open wound and not nosocomially acquired in the absence of any hardware.

The goal of treatment of chronic osteomyelitis aims to eliminate the pathogen and to debride necrotic bone [12]. Although the initial empiric antibiotic therapy often begins after obtaining appropriate cultures, the ultimate choice of antibiotic therapy relies on the bone culture and sensitivity pattern [1]. Our patient received two courses of TMP-SMX prior to surgical debridement and contributed to the controversy of the optimal route and duration of antibiotic therapy. In a small sample of twenty patients, Messina et al. observed a possible role of oral antibiotic therapy for acute osteomyelitis [15]. Data on the route and duration of oral antibiotic therapy for chronic osteomyelitis especially in the pediatric population are lacking [16]. In the absence of a consensus on the route of administration and duration of therapy, intravenous antibiotic therapy for two to six weeks before transitioning to oral antibiotics for a total of four to eight weeks has been recommended [1, 4]. Duration of therapy of six weeks is recommended to allow for osteogenesis [4]. Despite aggressive prolonged antimicrobial therapy and surgical debridement, relapse can occur as long as five years later [12].

## 5. Conclusions

We describe the first case of *S. schleiferi* chronic osteomyelitis in an immunocompetent adolescent. Our patient met the criteria for chronic osteomyelitis from the incomplete treatment of acute osteomyelitis. The uncommon pathogen *S. schleiferi* was after canine exposure and may be emerging in human infections. Thus far, data on the pathogenesis and virulence of *S. schleiferi* in various human conditions including osteomyelitis are sparse. Treatment strategies for *S. schleiferi* chronic osteomyelitis included prolonged antimicrobial treatment and a multidisciplinary approach, similar to the guidelines for *S. aureus* osteomyelitis. Healthcare providers need to be familiar with the increase of uncommon microorganisms such *as S. schleiferi* and their antibiogram when involving prolonged antimicrobial treatment and the potential risk of antibiotic resistance. Despite the immediate recovery we are reporting, prolonged follow-up will enhance our understanding and knowledge of this microorganism due to the high relapse rate of chronic osteomyelitis.

## 6. Limitations

We report a single case highlighting the identification of an uncommon pathogen in a known medical condition. This descriptive study was a retrospective review of electronic medical record notes written by physicians in training and specialists, laboratory findings, and imaging studies. Our conclusions were inferred. More data regarding *S. schleiferi* as a pathogen in humans are needed.

## Figures and Tables

**Table 1:** Reported cases of *Staphylococcus schleiferi* osteomyelitis.

No	Author/Year	Age (years)	Gender	Comorbidity	Source	Site	Treatment	Course (weeks)	Outcome
1	Calvo et al. 2000 [2]	67	Male	DM, AI	Surgery	Sternum	CLOXA, AMX	6	Recovered
2	Yarbrough et al. 2017 [3]	60	Female	Open wounds	Canine	Vertebra	VCM, CTRX	6	Recovered
3	Nguyen et al. 2020 [4]	75	Male	DM, IS	Bacteremia	Foot	VCM, CFPM, MTZ, TZP, CTRX	6	Recovered

AI: aortic insufficiency; DM: diabetes mellitus; IS: immunosuppressed. AMX: amoxicillin; CFPM: cefepime; CLOXA: cloxacillin; CTRX: ceftriaxone; MTZ: metronidazole; TZP: piperacillin-tazobactam; VCM: vancomycin.

## Data Availability

All data supporting this report are included in this report.
